# Mining Educational Data to Predict Students’ Performance through Procrastination Behavior

**DOI:** 10.3390/e22010012

**Published:** 2019-12-20

**Authors:** Danial Hooshyar, Margus Pedaste, Yeongwook Yang

**Affiliations:** Institute of Education, University of Tartu, Tartu 50103, Estonia; danial.hooshyar@ut.ee (D.H.); margus.pedaste@ut.ee (M.P.)

**Keywords:** educational data mining, predication of students’ performance, higher education, procrastination behavior, online learning

## Abstract

A significant amount of research has indicated that students’ procrastination tendencies are an important factor influencing the performance of students in online learning. It is, therefore, vital for educators to be aware of the presence of such behavior trends as students with lower procrastination tendencies usually achieve better than those with higher procrastination. In the present study, we propose a novel algorithm—using student’s assignment submission behavior—to predict the performance of students with learning difficulties through procrastination behavior (called PPP). Unlike many existing works, PPP not only considers late or non-submissions, but also investigates students’ behavioral patterns before the due date of assignments. PPP firstly builds feature vectors representing the submission behavior of students for each assignment, then applies a clustering method to the feature vectors for labelling students as a procrastinator, procrastination candidate, or non-procrastinator, and finally employs and compares several classification methods to best classify students. To evaluate the effectiveness of PPP, we use a course including 242 students from the University of Tartu in Estonia. The results reveal that PPP could successfully predict students’ performance through their procrastination behaviors with an accuracy of 96%. Linear support vector machine appears to be the best classifier among others in terms of continuous features, and neural network in categorical features, where categorical features tend to perform slightly better than continuous. Finally, we found that the predictive power of all classification methods is lowered by an increment in class numbers formed by clustering.

## 1. Introduction

The way in which students learn and teachers teach has changed considerably due to the rise of information and communications technologies in higher education. For example, online and blended courses’ (partially or fully) use of the Internet to deliver course content and instructions to learners, transform face-to-face learning into online learning [1]. Learning Management Systems (LMSs), which offer online learning materials, such as course content, quizzes, assignments, and forums, are considered as one way of supporting online learning. Teachers that use LMSs can simply manage and provide learning resources, and also monitor students’ learning progress as almost every action of the teachers and students in such systems are logged [2]. Gaining insight into the online behavior of students enables teachers to improve learning and teaching. However, it is worth mentioning that the data stored by LMSs is mainly raw and provides no solid information or measurements of existing theoretical concepts. Additionally, as many students using LMSs fail to adapt to the requirement of such environments, LMSs also create pedagogical challenges (besides their benefits) for teachers. Therefore, a better understanding of the process, and whether and how these data can be used for improving the learning process, is crucial [3,4].

There have been several studies revolving regarding the fundamental success or failure factors of online learning. For example, Azevedo et al. [5] and Hooshyar et al. [6] highlighted several challenges, e.g., self-pacing and self-regulation, more effort, rapid learning, etc., that usually hinder students from adapting to LMSs. Among all, procrastination—especially a tendency to put off completing tasks—and a lack of time are underlined by students as the main reasons when asked about their failure or dropping out of an online course (e.g., [7,8]). Tuckman [9] and Cerezo et al. [10] have stressed the negative effect of procrastination on learning achievement in students, whereas, similarly, Cerezo et al. [11] and Visser et al. [12] named procrastination as the most observed behavior in online learning which jeopardizes students’ academic success. Identifying students with learning difficulties in a course—and informing their teachers and themselves to take the necessary measures—is considered as one important step in improving student performance, leading to their academic success [13]. Educational Data Mining (EDM) is an emerging field focusing on developing methods that mine educational data to understand the behavior of students to possibly identify those with learning difficulties at early stages. The application of EDM techniques to educational data gives instructors the opportunity to take appropriate decisions which will eventually improve learning and lead to the improvement of academic success [14].

There has been extensive research employing EDM approaches to predict grades or the performance of students in a course. To do so, surprisingly, they mostly focus on students’ past performance (e.g., cumulative GPA) and/or non-academic factors (e.g., gender, age) to build their predictive models without considering students’ activity data [15]. Such predictive models in many cases simply fail to consider that many non-academic indicators (e.g., gender, race, socio-economic status) or past performance factors cannot be influenced by either students or teachers. In many situations, these models may negatively affect students’ performance and discourage them—if students are made aware that such variables are used in the prediction of their performance—because they may assume that their past circumstances have already set them up for (future) failure [16]. Instead, more research should use students’ activity data—which can, logically, be among the best indicators of students’ performance and course grade—during the course to develop their predictive models.

Given the importance of procrastination as an underlying indicator (which is related to students’ activity and performance in a course, not their past performance) for predicting students’ performance in a course and the success of advanced EDM approaches in predicating students’ performance, there are only a very few studies that have taken into account the application of EDM techniques for prediction of students’ performance in a course through their procrastination behaviors. Yet, though the related research shows good performance and has proven to be useful (e.g., [17]), they ignore some important factors, for example, including underlying factors of procrastination behaviors (e.g., inactive time which is the time that goes by from when assignments become open until students view it for the first time), employing and comparing advanced EDM approaches, and considering practitioners by proposing simple and easy-to-implement EDM approaches which are yet precise, indicating a research gap. Research filling this gap could shed a light on useful means for enhancing EDM research in higher education.

In this work, to address these issues, we propose an algorithm, called PPP, for predicting students’ performance through procrastination behaviors using their assignment submission data. To do so, we first build feature vectors representing the submission behavior of students for each assignment, using inactive and spare time. We then use clustering and classification methods to put students into different procrastination behavior categories based on their submission behavior. As both types of quantitative variables are usually used in building feature vectors in EDM research (continuous and categorical), we consider both types of variables to investigate which works best in PPP. To this aim, we formulate our research question as follows:

- How accurately can our proposed algorithm predict students’ performance through their procrastination behaviors?
- Which classification method offers superior predictive power, when using various numbers of classes in the feature vectors? What is the effect of using continuous versus categorical feature vectors on different classification methods?

Our proposed algorithm contributes to EDM research in multiple ways: (1) It predicts students’ performance through their procrastination behavior using their submission data; (2) it considers fundamental variables of students’ procrastination behaviors, including students’ behavioral patterns, before the due date of assignments, to establish a comprehensive students’ submission behavior; (3) it takes into account the benefits of cluster analysis to semantically ensure the appropriateness of the clusters to the procrastination behavior of students; and (4) it takes into account both continuous and categorical feature vectors to study the effect of different feature types of prediction power of our algorithm.

The outline of this paper is as follows: Section 2 reviews the related studies in the area of academic procrastination, EDM research, and its application in a procrastination context. Section 3 lays out our proposed methodology. Section 4 revolves around the experimental results. Finally, Section 5 and Section 6 offer discussions and the conclusion of this study, respectively.

## 2. Previous Research

### 2.1. Academic Procrastination

Procrastination is defined as “intentionally delaying or deferring work that must be completed” by Schraw et al. [18] (p. 12). In general, procrastination is the lack of willingness and intention to perform an activity or the tendency to postpone an activity to the last possible minute, which is actually the opposite of motivation [19]. Several studies reported that up to 29% of the adult population are chronic procrastinators and procrastination tendency is a widespread phenomenon amongst western societies [20,21].

Students, similarly, demonstrate such behavior while performing academic tasks [7]. This can result in both negative and positive consequences. According to Chu and Choi [22], procrastinators can be divided into two groups, active and passive. Active procrastinators deliberately procrastinate as a positive academic strategy because they prefer to work under pressure, whereas passive procrastinators allow the negative, indecisive behavior to disable them, causing them to suffer from negative academic consequences. Steel [23] likewise discusses that procrastination can be described as a positive behavior and some researchers even refer to it as “functional delay” (p. 66). Nevertheless, he concluded, in his meta-analysis of the literature of procrastination, that positive referral of procrastination is secondary compared to the traditional negative sense. In this study, therefore, the term procrastination only refers to its primary, passive, negative form. Previous research on procrastination was more inclined toward studying the relations between psychological factors or personal traits and procrastination. Ackerman and Gross [24] and Van Eerde [25], for example, highlighted personality traits, performance outcomes, and emotional dispositions as underlying factors for procrastination. Nonetheless, many more studies correlated procrastination tendencies to time management (e.g., [10,26]). Visser et al. [27], for instance, state that there is a relation between fear of failure and time management, Hen and Goroshit [28] correlate time management to anxiety and stress, and, finally, Balkıs [29] and You [30] highlight the effect of time management on academic achievement.

Multiple studies negatively correlated procrastination to academic performance and reported that this tendency usually leads to negative results, such as a decrease in course achievement and long-term learning, lower goal commitment and grade, and many more. For instance, Cerezo et al. [11] concluded that student achievement is, to a large extent, negatively linked to the procrastination tendency, and in a similar vein Michinov et al. [8] reported the negative effect of procrastination tendency on course achievement. On the one hand, some existing research found a negative relationship between academic achievement in conventional learning environments and procrastination [31,32]. According to the findings from Melton’s [33] study, students who postponed their academic tasks to the very last moment demonstrated lower retention of learning materials in the long-term as compared to those who studied regularly. Additionally, the time required to complete the task was mostly underestimated by chronic procrastinators and not enough time was allocated to complete the task, causing the students’ failure most of the times [26]. On the other hand, numerous studies highlight that the dropout rate of online students compared to traditional learning environments seem to be more related to procrastination behavior and such a tendency can be more detrimental (e.g., [10]). One reason could be the fact that learning depends more on the individual learner. To this end, Elvers et al.’s [34] study on individual behavior of students in online course found that students mostly access or visit webpage of courses right on the exam day or the day before. The authors further argued that students do not follow course schedules and simply put off their academic tasks until the last moment. A similar finding has been highlighted by many more studies and time management has been frequently named as a solid indicator of academic achievement [8,35]. For instance, Wighting et al. [35] concluded that a delay in performing tasks is one of the significant predictors of engaged learning, leading to procrastination. Finally, according to findings reported by Tuckman’s study [9], non-procrastinators in online environments tend to perform better than procrastinators, and procrastination behavior is much stronger in an online learning environment compared to a traditional learning environment. Consequently, it is essential to detect such tendencies in a timely manner in learning environments. Self-reported questionnaires have been used, rather than observed behaviors, by several studies to measure the procrastination behavior of students (e.g., [8,35]). An alternative way, however, is to obtain students’ learning behavior in a timely manner by using their log data in the system.

### 2.2. Educational Data Mining

The process of converting raw educational data into useful information that could have a further great impact on educational research and practices is called Educational Data Mining (EDM). EDM approaches, in many different ways, provide instructors and students with useful insight into their learning process so they can take appropriate actions and decisions which will eventually improve learning achievement and lead to an improvement of academic success [14]. These include offering feedback, generating recommendations, prediction of learning difficulties, offering personalized learning, etc. Researchers usually apply different data mining techniques, such as clustering and classification, to educational context to discover hidden knowledge and patterns. A systematic review conducted by Dutt et al. [36] provides a comprehensive review of EDM research. In the following subsections, we give a broad overview of the two methods, clustering and classification, used in this study.

#### 2.2.1. Clustering Methods in the Context of Education

Clustering is the process of dividing data points into a number of groups where similar ones are partitioned together. Several clustering methods have been applied to various variables within the context of education, providing an unambiguous schema of students’ learning styles according to various variables, such as time spent on completing learning tasks, learner behavior, student motivation, etc. For example, Tair and El–Halees [37] employed various clustering methods to cluster students’ performance, using student information from 1993 to 2007, with the aim of improving students’ performance and overcome the problem of low grades. In a different vein, Li and Yoo [38] applied clustering methods on a dataset of 89 students’ interactions with an adaptive tutoring system to model students’ e-learning behavior for further adaptive and effective teaching in the context of a CS-1 course. Pedaste and Sarapuu [39] applied clustering on the level of student teams at the beginning of the learning process and personalized learning based on the characteristics of clusters and achieved a significant improvement in problem solving skills. In their study hierarchical cluster analysis was used and the number of meaningful clusters was detected by humans manually. Even though Dutt et al. [36] in their systematic study indicated that *k*-means was one of the most reliable methods which had been employed by many researchers, several other studies named spectral clustering as one of the most popular modern clustering methods in data mining that usually performs better than traditional clustering methods (including *k*-means) [40,41]. Clustering methods, such as spectral clustering, sometimes do not specify the number of clusters, requiring manual specification of the number of clusters. As this number is often unknown, several trials of the *k* value are needed until a good balance is found where larger and smaller values might result in clusters that are too detailed and coarse, respectively. In such cases, the optimal number of clusters can be identified by various approaches, among them the Elbow method. We, in the present study, employ the spectral clustering method.

#### 2.2.2. Classification Methods in the Context of Education

Classification is a frequently used data mining method in education context, assigning an object to a class. In other words, classification is a specific case of prediction where a classifier—which uses training data to produce a classification model—predicts a class (label) or a discrete value [42,43,44]. Classification methods have been widely used in education to classify students according to their motivation, knowledge, and behavior (e.g., [45]). For example, Ahmad et al. [46] predicted, using eight-year data from 2006 to 2014, students’ academic performance of undergraduate students in computer science courses. The dataset used contains the students’ demographics, previous academic records, and family background information. They employed various classification techniques, including Decision Tree and Naïve Bayes, for this prediction. Kotsiantis et al. [47] trained six classification algorithms on a dataset from the Hellenic Open University for identifying poor performers in a distance learning environment. They concluded that the Naïve Bayes algorithm is the most appropriate to be used for the construction of educational software support tools. Additionally, Huang and Fang [48] reported that support vector machine (SVM) performed best in predicting student academic performance in an engineering dynamics course. Among different classification methods for prediction, decision trees, SVMs, neural networks, Bayesian classifiers, and nearest neighbor techniques are among the best-known classification paradigms [49].

### 2.3. Procrastination Prediction Using EDM Methods

Several researchers have carried out studies to predict performance of student through different variables using EDM methods (see [15]). Nonetheless, students’ activity data including homework submission behavior of students—which, logically, can be among the best indicators of students’ performance and course grade—to a large extent has been ignored.

To this end, there exist insufficient studies that have taken into account the application of EDM techniques for the prediction of students’ performance or success in a course through their procrastination behaviors. Table 1 briefly shows the comparison of related works in the area. We also provide in the table an overview of the approach provided in our work to show how the existing approaches could be improved. For instance, Drăgulescu et al. [17] used variables before and after the submission of assignments to predict students’ assignment submissions. To predict students’ submissions in a specific time segment, they used data from that time segment and prior segment attributes. Similar to other existing works, they considered those assignments submitted before the deadline as on-time and those after as late submissions, ignoring the actual students’ behavioral patterns before the due date of homework in the decision-making of their proposed approach (for example, one can submit his/her assignment on-time, but be a candidate for future procrastination). Additionally, they ignored using and comparing advanced classification methods in their model. In a similar attempt, Akram et al. [50] proposed a prediction model to predict the academic performance of students through their homework submission behavior. Even though their approach is novel and proved to be successful, similar to Drăgulescu et al. [17]’s work, variables used for procrastination tendencies were limited. Furthermore, they compared various classification methods to find the best, but they had not considered advanced classification approaches, such as SVM, neural networks, etc.

Finally, Olivé et al. [51] proposed a generalizable predictive model for different courses which uses neural networks. They predicted the likelihood of student assignment submissions being on time, in which students would likely submit their assignments on time, based on their activity up to two days before assignments’ due dates. Even though they achieved good accuracy in their models and they had considered some of procrastination-related variables in their input features (as well as many more), their proposed model suffers from high complexity and is thereby difficult to implement, interpret, and use by practitioners. This issue becomes more apparent when looking for the reasoning behind the decision made by the model. Additionally, they did not consider comparing their proposed approach with other existing classification methods that usually function well (with less data) and can be implemented and interpreted easier. Research filling the abovementioned gaps could shed light on useful means for enhancing EDM research in higher education.

## 3. Method

### 3.1. Problem Description

Let us assume that we have a set of students denoted as $S=\left\{s_{1},\;s_{2},\ldots ,\;s_{n}\right\}$ that are expected to submit a set of assignments denoted as $A=\left\{a_{1},\;a_{2},\ldots ,\;a_{m}\right\}$ within a deadline. Some students may submit their assignment on time, some may submit with delay, or, finally, some may never submit. Each student *s* is associated with a number of dates of first assignment view $FirstviewD^{s}$ = ($FirstviewD_{1}^{s},FirstviewD_{2}^{s},\;\ldots ,FirstviewD_{m}^{s}$) and the number of the assignment submission $SubmissionD^{s}$ = ($SubmissionD_{1}^{s},SubmissionD_{2}^{s},\ldots ,SubmissionD_{m}^{s}$), where $FirstviewD_{m}^{s}$ and $SubmissionD_{m}^{s}$ denote the *m-*th assignment first view and assignment submission of student *s*. Each assignment a is associated with an open date $OpenD^{a}$ and a deadline $Deadline^{a}$. All students’ $FirstviewD^{s}$ and $SubmissionD^{s}$, and assignments’ $OpenD^{a}$ and $Deadline^{a}$ are used to build spare time—time that goes by from when a student submits assignments until the assignment is due—and inactive time—time that goes by from when assignments become open until a student views it for the first time—for each student. This gives us the opportunity to have information on a student’s procrastination behavior. We seek, according to such information, to predict if a student is a procrastinator, a procrastinator candidate, or a non-procrastinator by considering if they will have submitted their assignment before the deadline and the time they will have missed before they actually start working on their assignment. Table 2 shows notations used in this work.

### 3.2. PPP: Prediction of Students’ Performance through Procrastination Behavior

**Figure 1:**
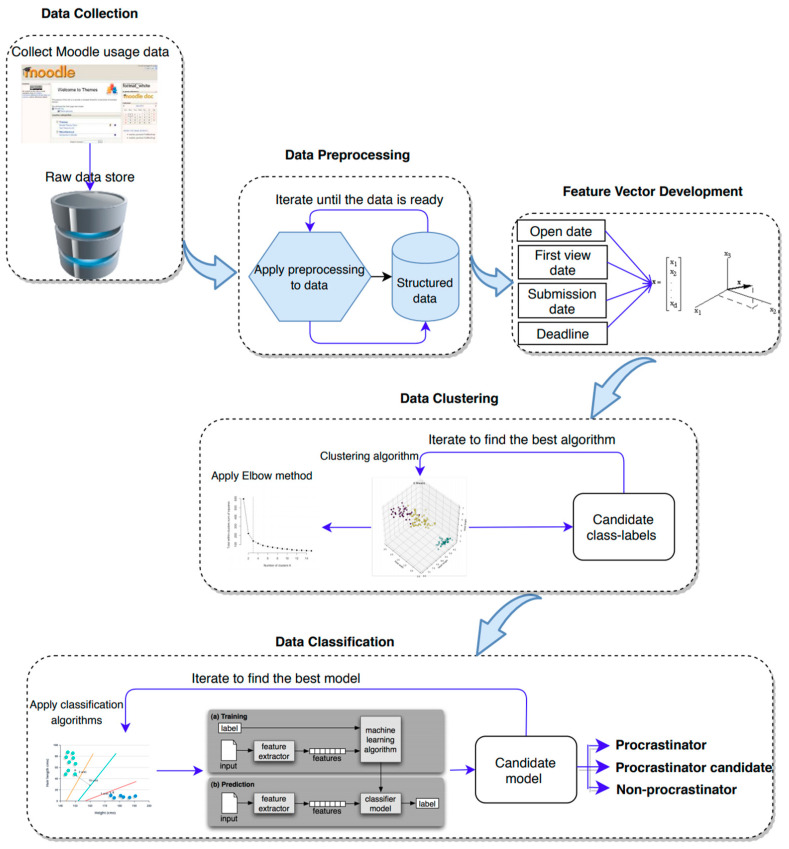
Framework of the PPP approach.

In this section, we explain our proposed approach to predict students’ procrastination behavior using their submission data. Figure 1 gives a summary of the approach and subsequent subsections elaborate each step of the approach. The proposed framework consists of five steps. First, data needs to be collected. In our case it was extracted from Moodle. Next, data preprocessing is needed to structure it appropriately for the third phase, which is feature vector development. In this step all subjects (learners in our case) will be characterized mathematically by combining information available about the objects (assignments in our case). The fourth step would be to cluster students with similar behavioral categories by means of a clustering technique and, in the final step, the data will be classified according to this and predictions will be made regarding the subjects (the procrastination behavior of students).

#### 3.2.1. Building the Feature Vector of Assignment Submission Behavior

We present each assignment by continuous and categorical features, where each include a pair of continuous and categorical values (see Equations (1) and (2)). This enables us to investigate both types of quantitative variables and possibly determine which works the best in our proposed approach. We present in Equations (3) and (4) each student by a feature vector for all assignments:

(1) $$x_{i}=(v_{1},\;v_{2})$$

(2) $$y_{i}=(w_{1},\;w_{2})$$

(3) $$X_{j}=(x_{1j},\;x_{2j},\ldots x_{ij})$$

(4) $$Y_{j}=\left(y_{1j},\;y_{2j},\ldots y_{ij}\right)$$

We show in Algorithm 1 the process to compute $v_{1}$ and $v_{2}$, as well as the feature vector *X*. From our dataset the algorithm inputs OpenD, FirstviewD, SubmissionD, and Deadline. For each student, values of $v_{1}$ and $v_{2}$ are then computed for each assignment, where *i* and *j* are the total number of assignments and students, respectively. In the next step, it is decided whether the spare time should be flagged with 0 or 1, indicating whether the assignment submission is on time or late (or non-submission). In the final step, inactive time for each assignment is considered and, using the median of inactive time, inactive time for an assignment is flagged with a 0 or 1, indicating small or large amounts of inactive time for each student for each assignment. The output of this algorithm is the feature vectors ***X*** and ***Y***.
**Algorithm 1** Development of feature vectors ***X*** and ***Y***Input: $OpenD^{a},\;FirstviewD^{a},\;SubmissionD^{s},\;Deadline^{s}$**,*** S, A*Output: Feature vector ***X*** and ***Y***1: Initialize *j* = |*S*|, *i* = |*A*|2:  while *n < j* do3:    while *m < i* do 4:     $x_{nm}\left[v_{1}\right]=\frac{Deadline^{m}–SubmissionD_{m}^{n}}{Deadline^{m}–OpenD^{m}}$
5:     $x_{nm}\left[v_{2}\right]\;=\;\frac{FirstviewD_{m}^{n}–\;OpenD^{m}}{Deadline^{m}–\;OpenD^{m}}$
6:     if $x_{nm}\left[v_{1}\right]$
*< =* 0 then7:       $y_{nm}\left[w_{1}\right]$ = 0 8:     else9:       $y_{nm}\left[w_{1}\right]$ = 110:     if $x_{nm}\left[v_{2}\right]$
*= >* median $x_{nm}\left[v_{2}\right]$ then11:       $y_{nm}\left[w_{2}\right]$ = 012:     else13:       $y_{nm}\left[w_{2}\right]$ = 1 14:     end if15:   end while16: end while 17: return Feature vector ***X*** and ***Y***

#### 3.2.2. Finding the Optimal Number of Classes Using Clustering

Clustering is the process of dividing data points into a number of groups where similar ones are partitioned together. In Algorithm 1 we described a novel process to build assignment submission feature vectors for each assignment. Outputs of this algorithm are further used to group students with similar behavioral categories by means of a clustering technique. Different numbers of clusters help to detect the students with learning difficulties, and adds a class-label to the feature vectors.

The spectral technique has been employed successfully by many researchers in data mining (e.g., [40]), as it is one of the most popular modern clustering methods in data mining. The spectral technique does not specify the number of clusters, requiring manual specification of the number of clusters. As this number is often unknown, several trials of the *k* value are needed until a good balance is found where larger and smaller values might result in too detailed and coarse clusters, respectively. In such cases, the optimal number of clusters can be identified by various approaches, among them the Elbow method. In this method, after computing the clustering methods for different k values, a distortion score is calculated, which is the sum of the square of the distances from each point to its assigned center. The suitable number of cluster is then specified by a bend (knee) location in the plot. We, in the present study, employ spectral clustering and the Elbow method to find the optimal number of clusters. However, before application of the Elbow method to find the optimal number of clusters, we statistically analyze different number of clusters generated by the clustering algorithm (*k* = 2, 3, and 4). Algorithm 2 illustrates the process of spectral clustering, finding the optimal number of clusters, and validation (further analysis) of the optimal number of clusters.
**Algorithm 2** Discovering the optimal number of clusters using the Elbow methodInput: feature vectors (outputted from algorithm 1) without class labels, the maximum number of clusters *k*Output: (validated) optimal number of clusters1:  while i<k do2:    Construct a similarity graph and let ***W*** be its weighted adjacency matric3:    Compute the unnormalized Laplacian *L*4:    Compute the first k eigenvectors $u_{1},\ldots ,\;u_{k}$ of the generalized eigenproblem Lu = λDu
5:    Let $U\in {\mathbb{R}}^{n*k}$ be the matrix containing the vectors $u_{1},\ldots ,\;u_{k}$ as columns 6:    For i=1, …, n let $y_{i}\in {\mathbb{R}}^{k}$ be the vector corresponding to the *i*-th row of ***U***7:    Cluster the points ${\left(y_{i}\right)}_{i\;=\;1,\ldots ,n}$. in ${\mathbb{R}}^{k}$ with the *k*-means algorithm into clusters $C_{1},\;\ldots ,\;C_{k}$
8:    Calculate distortion score9: end while10: Plot the curve of distortion score according to the number of clusters *k*
11: Consider the location of a bend (knee) in the plot as the optimal number of clusters 12: Validate the optimal number of cluster through further (statistical) analysis of different number of clusters 13: return the optimal number of clusters

#### 3.2.3. Classification of Students Using Class Labels

Once the best set of clusters is found by the clustering method (outputted from Algorithm 2), a predictor is trained to classify students into different classes. To do so, various classification methods are compared to find the most suitable classifier for predicting students’ procrastination behavior. These include linear and radial basis function kernel support vector machines (L-SVM and R-SVM), Gaussian processes (GP), Decision Tree (DT), Random Forest (RF), Neural Network (NN), AdaBoost (ADB), and Naive Bayes (NB). These classification methods are selected due to their popularity—the fact that they have been successfully used by many researchers in EDM research—and high performance compared to traditional methods (e.g., [36,44]). The classification methods with different numbers of classes are then compared by means of four different evaluation measures, namely accuracy, F1-score, precision, and recall. This process, along with the generalized algorithm of PPP, is illustrated in Algorithm 3, where 5-, 10-, 15-, and 20-fold cross-validation is used in classification, dividing the data into two parts with the purpose of statistically comparing and evaluating the learning algorithms. In other words, different *k-*fold (i.e., 5, 10, 15, and 20) is used to show the stability of the models. Observe that during the experiment the training and test set division will remain constant.
**Algorithm 3** PPP: Prediction of students’ performance through procrastination behaviorInput: $OpenD^{a},\;FirstviewD^{a},\;SubmissionD^{a},\;Deadline^{a}$*, S, A*Output: prediction of procrastination behavior (if a student is procrastinator, procrastinator candidate, or non-procrastinator) 1: Implement algorithm 1 to build feature vector ***X*** and ***Y***
2: Implement algorithm 2 to produce the optimal number of clusters from the feature vector3: Apply classification algorithm using class labels4:      L-SVM, R-SVM, GP, DT, RF, NN, ADB, and NB 5: Compare classification algorithm performance by using test data6:      $P_{c}\;=\;P_{1},\;P_{2},\;P_{3},\;\ldots ,\;P_{n}$7:  while *i* < = *n* do8:    if $P_{ci}\;>\;P_{ci+1}$ then 9:     $C\leftarrow c_{i}$. 10:    else11:     $C\leftarrow c_{i+1}$12:    end if13: end while14: Choose the best performed classification algorithm C15: Employ the classification C to predict procrastination16: return prediction of procrastinator, procrastinator candidate, or non-procrastinator

## 4. Experimental Results

### 4.1. Dataset

We used in our experiment activity data of students extracted from the University of Tartu’s Moodle system which usually logs performed students’ activities. The blended course that we collected data from was entitled “Teaching and Reflection” which is taught as a compulsory course for all teacher education students in the Institute of Education. Basically, blended courses use Moodle as a means for interaction beyond classroom. More specifically, instructors design their courses using the Moodle platform—where they divided the courses into several modules where there exist various types of resources (learning materials), tasks (usually quizzes), and assignments related to each module—and ask students to perform various online activities. For each assignment, there is an opening time (an instructor may create a course well before the beginning of a semester but opens it up to students shortly after the semester has started) and a deadline specified by the instructor where students have to submit or upload their assignment.

### 4.2. Label

We extracted four variables from the logs of the courses, including the open date of an assignment (OpenD), the date of first view of the assignment (FirstviewD), the date of assignment submission (SubmissionD), and the due date of the assignment (Deadline). We created two datasets using the variables for further analysis, listed in Table 3.

### 4.3. Results

Before using a clustering technique in our approach, however, it is required to present descriptive statistics of spare time ($v_{1}$), inactive time ($v_{2}$), and assignment scores (see Table 4). Table 4 reveals a positive correlation between spare time and assignment scores in the course. Nonetheless, there exists a negative link between assignment score and inactive time.

#### 4.3.1. Phase 1: Clustering Development and Analysis

**Figure 2:**
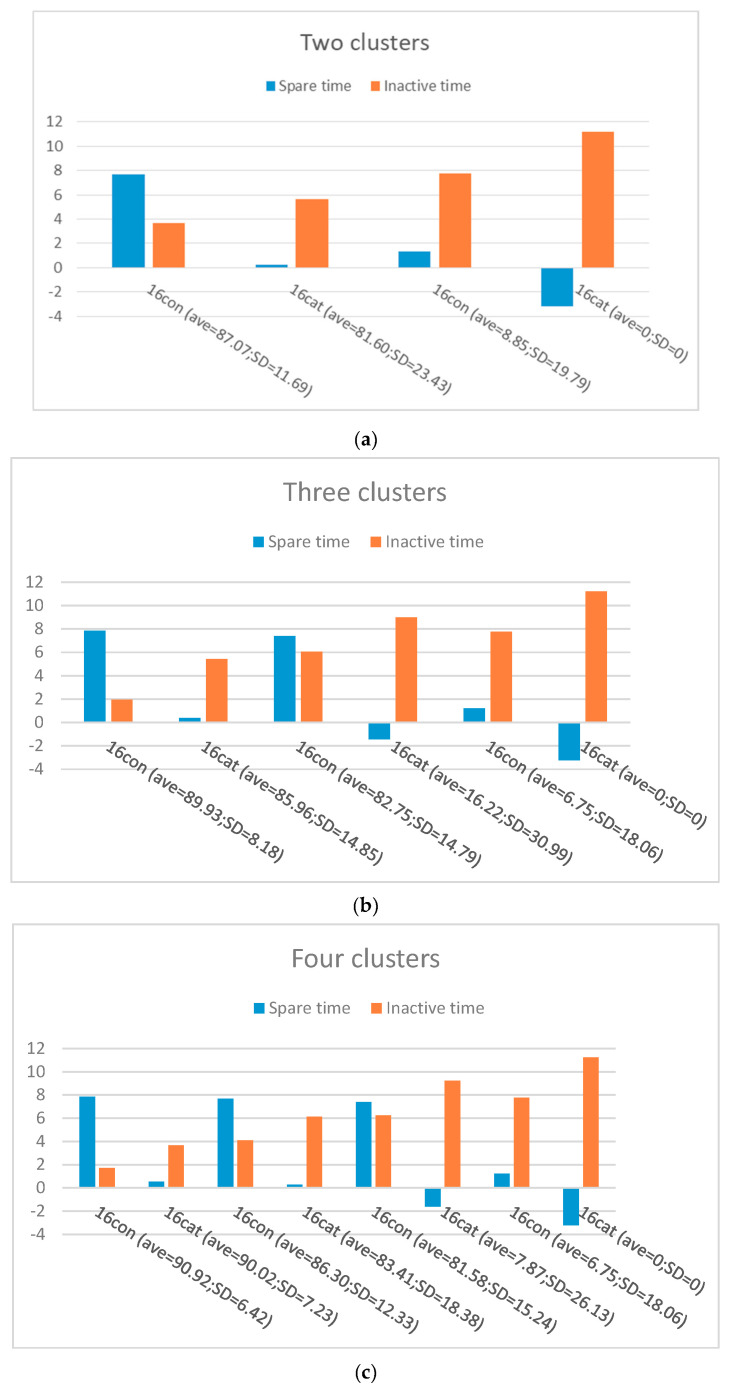
Clusters produced by the spectral method: (**a**) *k* at 2, (**b**) *k* at 3, and (**c**) *k* at 4.

As mentioned previously, to decide the correct number of classes of students with similar behavioral categories, we employed the Elbow method along with further (statistical) analysis. Figure 2 shows the mean and standard deviation of the clusters (both $v_{1}$ and $v_{2}$) produced by the spectral method with different values of *k* = 2, 3, and 4.

Figure 2a illustrates two different clusters for the feature vectors: Cluster A with a small inactive time and relatively large spare time, and Cluster B with a larger inactive time and a smaller spare time than cluster A. Those in Cluster A are considered as non-procrastinators with a high average score (average = 85.95 and SD = 14.68), while those in Cluster B are considered as procrastinators with a lower average score (average = 24.61 and SD = 18.21). Figure 2b shows three different clusters, for the feature vectors, when the value of *k* is at 3. Cluster A has a higher average score (average = 88.48 and SD = 10.16), smaller inactive time, and a relatively larger amount of spare time compared to Clusters B and C, so we consider them as a group of non-procrastinators. Cluster B (average = 61.45 and SD = 20.24) appears to have a lower and higher average scores than Clusters A and C, respectively. Additionally, inactive times are almost larger than in Cluster A and smaller than in Cluster C, whereas spare times are relatively larger than in Cluster C and smaller than in Cluster A. Those in this cluster can be regarded as procrastinator candidates. Finally, those in Cluster C, with a lower average score (average = 6.75 and SD = 15.62) than the other two, can be considered as procrastinators.

Figure 2c shows four different clusters, for the feature vectors, when the value of *k* is at 4. As it can be seen, Cluster A (average = 90.1 and SD = 7.21), B (average = 86.3 and SD = 12.84), and C (average = 56.82 and SD = 20.56) appear to have high average score, whereas cluster D has a very low average score (average = 2.25 and SD = 6.02). Clusters A and B, with small inactive times and larger spare times compared to other clusters, and higher average scores can be considered as non-procrastinators. Cluster D, with the lowest average score, has the greatest inactive time and smallest spare time than the other three clusters and can be seen as procrastinators, while Cluster C with a relatively high average score, large inactive time, and small spare time can be called procrastinator candidates. This result shows that, overall, as the average assignment score decreases, the inactive time tends to increase, while spare time has a tendency to decrease.

**Figure 3:**
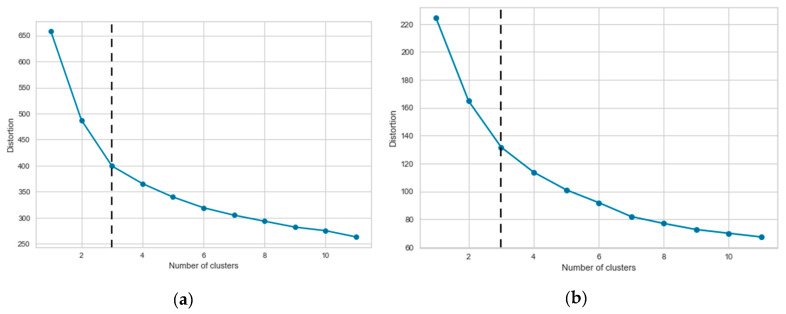
Elbow result: (**a**) Continuous features, and (**b**) categorical features.

In Figure 3, using the Elbow method, the optimal number of clusters are shown. More specifically, Figure 3a,b shows the elbows for continuous and categorical features, respectively. As the figure demonstrates, the optimal number of clusters for both continuous and categorical features is three. We thus decide, according to our cluster analysis—that showed no distinct group can be formed beyond three—and the Elbow result, to consider three as the optimal cluster number.

#### 4.3.2. Phase 2: Classification

We applied in this study eight different classification methods to classify the data. We then compared the performance of the classification methods—produced by L-SVM and R-SVM, GP, DT, RF, NN, ADB, and NB—with three different numbers of classes. Regarding the parameters used in the classification methods in our experiments, we set the regularization parameter *C* = 0.025, degree *d* = 3, and learning rate ε = 0.001 for L-SVM; *C* = 1.0, degree *d* = 3, and learning rate ε = 0.001 for R-SVM; kernel = 1.0 × RBF (1.0) for GP; maximum depth = 5, minimum samples split = 2, and minimum samples leaf = 1 for DT; number of estimators = 10, maximum depth = 5, and minimum samples split = 2 for RF; hidden layer size = 100, activation function = ‘relu’, adam optimization, and learning rate ε = 0.001 for NN; number of estimators = 50 and learning rate = 1.0 for ADB; and, finally, smoothing = 1 × 10^−9^ for NB. We used accuracy, F1-score, precision, and recall as performance metrics to evaluate the classification techniques. Table 5 lists the average of all performance metrics at different *k*-fold for all classification methods.

According to Table 5, in two-class with continuous and categorical features, L-SVM and R-SVM with 99% accuracy show superior performance, respectively. In regard to three-class, L-SVM likewise shows the best performances with 95% accuracy in continuous features. However, NN with 96% accuracy, outperformed other methods with categorical features. In four-class with continuous and categorical features, similar to three-class with categorical features, NN shows a better performance with 88% accuracy compared to other methods.

**Figure 4:**
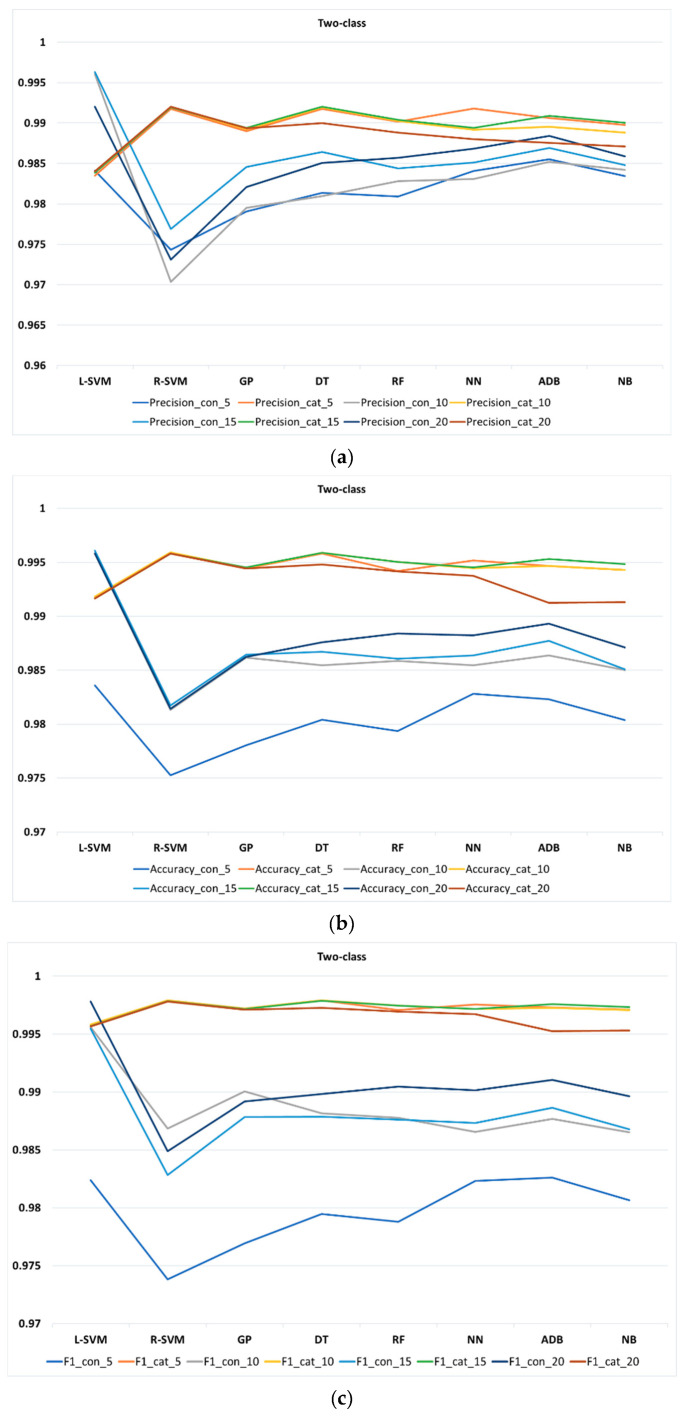
Performance metrics of classification methods at different *k*-fold for two-class: (**a**) precision, (**b**) accuracy, and (**c**) F1-score.

**Figure 5:**
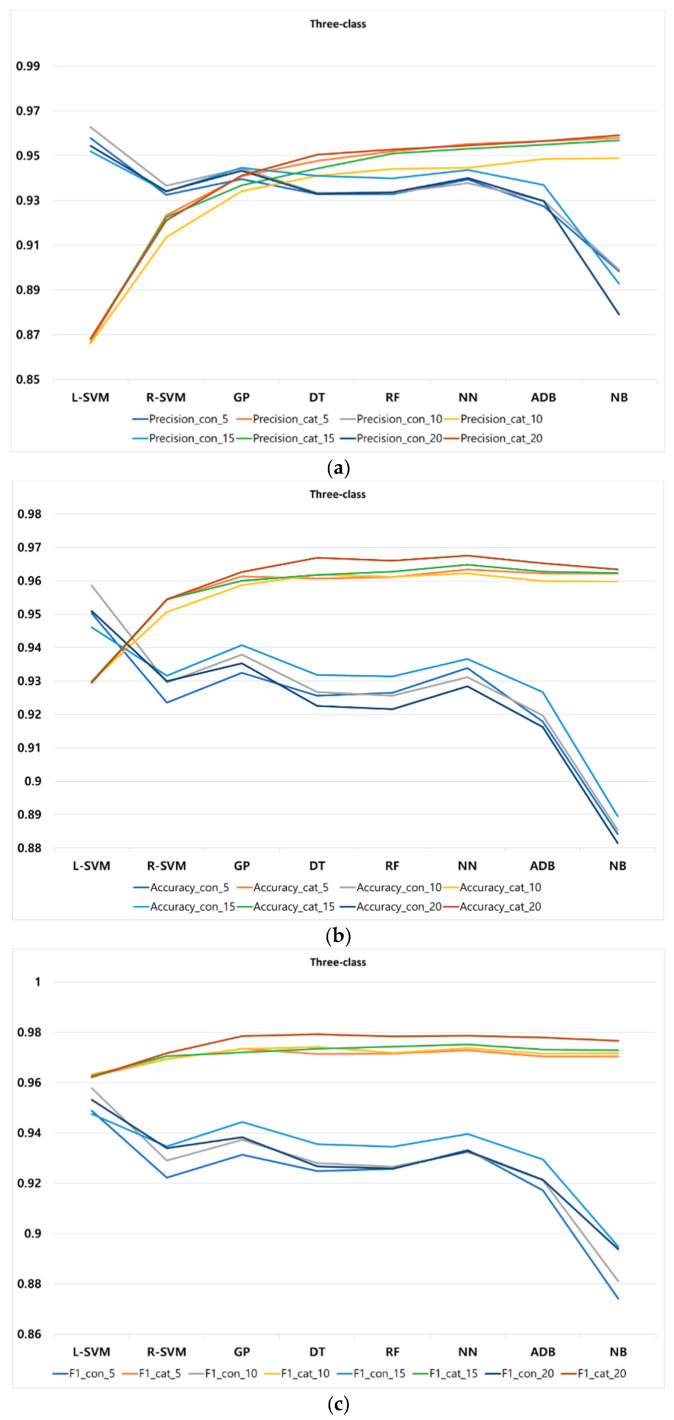
Performance metrics of classification methods at different k-fold for three-class: (**a**) precision, (**b**) accuracy, and (**c**) F1-score.

**Figure 6:**
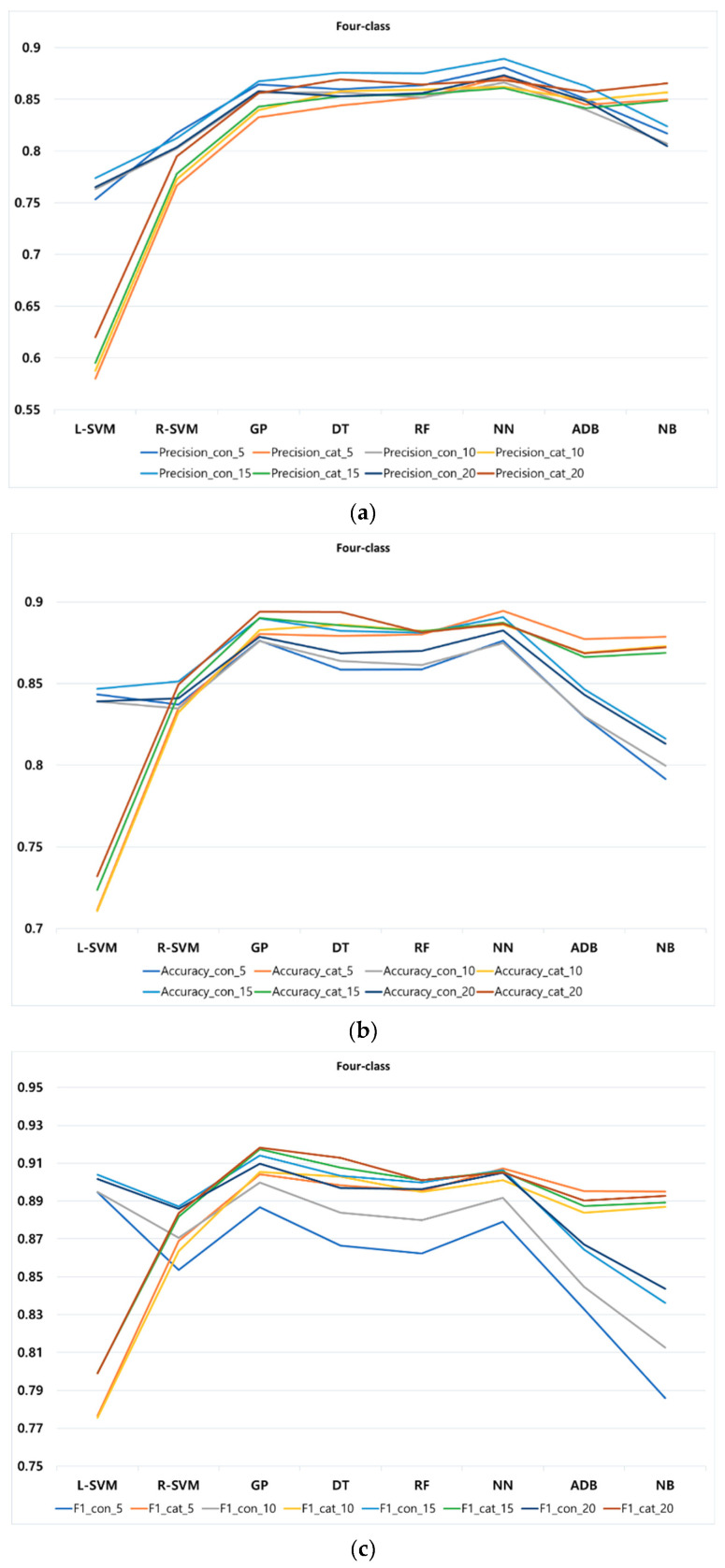
Performance metrics of classification methods at different *k*-fold for four-class: (**a**) precision, (**b**) accuracy, and (**c**) F1-score.

Additionally, Figure 4, Figure 5 and Figure 6 display different *k*-fold for all performance metrics (namely, precision, accuracy, and F1-score) of the classification methods in two-, three-, and four-class, respectively. Table A1 in the Appendix A lists the values of all performance metrics of the classification methods in three classes. Observe that due to the high similarity between the result of recall and accuracy, we decided not to produce figure to show the result of recall.

As Figure 4a shows, in two-class, L-SVM appears to have a higher precision with continuous features than other methods in different *k*-folds, whereas R-SVM shows the best precision with categorical features in different *k*-folds. Figure 4b,c shows the same for accuracy and F1-score in two-class, with L-SVM and R-SVM performing the best among other methods in continuous and categorical features (at different *k*-folds), respectively. Furthermore, different values of *k*-fold result in a slight increment or decrement of precision, accuracy, and F1-score in all classification methods.

In three-class, according to Figure 5, L-SVM shows the highest precision with continuous feature among all classification methods at different *k*-fold. However, unlike two-class categorical features, NB has the best precision using categorical features at different *k*-fold. Regarding the accuracy of three-class, the best performance in continuous and categorical features belong to L-SVM and NN at different *k*-fold, respectively. Lastly, concerning F1-score, L-SVM and NN likewise show a better performance with continuous and categorical features compared to other methods. Regarding performance of classification methods at different *k*-fold, similar to two-class, different values of *k*-fold cause a slight increment or decrement of precision, accuracy, and F1-score in all classification methods, showing the stability of our proposed approach.

In four-class, according to Figure 6, NN in several cases outperforms other methods in continuous and categorical features when it comes to precision and accuracy at different *k*-fold. However, in F1-score, GP almost outperforms all other methods in both continuous and categorical features at different *k*-fold. Similar to two- and three-class, different values of *k*-fold lead to a slight increment or decrement of precision, accuracy, and F1-score in all classification methods.

Considering our findings shown in Table 5 and Figure 4, Figure 5 and Figure 6, it can be concluded that L-SVM and R-SVM are the best classification methods in two-class, at different *k*-fold, with continuous and categorical features, respectively. In terms of three-class, our results highlight L-SVM and NN as the best performed methods at different *k*-fold for continuous and categorical features, respectively. Finally, in four-class, NN mostly performs the best in both continuous and categorical features at different *k*-fold. Regarding employing different number of *k*-folds on the methods, we found that, in our proposed approach, most of the methods tend to be rather stable using different *k*-folds. This means in both situations—when differences between training and test set size is large or rather small—classification methods appear to be stable in our approach regardless of their bias.

## 5. Discussion

We proposed in this study a novel algorithm for automatic assessment of students’ performance through procrastination behaviors by using their assignment submission data (called PPP). PPP can, in a timely manner, predict and identify students facing learning difficulties at the beginning of the semester and provide educators with an opportunity to intervene when necessary (by taking necessary remedial actions). For example, educators can keep those students that are flagged procrastinators or procrastination candidates by PPP under observation and provide them with further assistance in completing their assignments. More interestingly, instructors can offer feedback to all three groups of students—procrastinators, procrastination candidates, and non-procrastinators—according to PPP. Offering timely feedback (produced through different means, such as the prediction of students’ future behaviors and learning difficulties) to students rather than giving feedback at the end of semester is an important factor in their academic performance. As reported by many researchers, e.g., [52,53], regular, timely, and appropriate feedback plays an important role in both reducing procrastination tendencies and boosting students’ performance. Michinov et al. [53] stated that informing students about their peers’ performance could potentially reduce their procrastination tendencies, whereas Tuckman [52] noticed that encouraging and motivating students could lead to decreased procrastination tendencies.

PPP has been designed and developed to automatically detect different type of procrastination behaviors and could be considered as a generalized approach, making it a good candidate for providing personalized learning for students with different needs. To develop PPP, we firstly, in a novel way, calculated spare time and inactive time—time that goes by from when a student submits the assignments until the assignment is due and time that goes by from when assignments becomes open until a student views it for the first time, respectively—for developing feature vectors which represent students’ submission behaviors. It should be noted that, unlike many existing works that ignore the actual students’ behavioral patterns (for example, one can submit his/her assignment on-time, but be a candidate for future procrastination) before the homework due date in the decision-making of their proposed approach, PPP takes into account on-time, late, or non-submissions, and the students’ behavioral patterns before the homework due date to have a comprehensive students’ submission behavior during the whole semester.

Secondly, using the feature vectors and Algorithm 2, we clustered students with similar behaviors in different groups, namely procrastinators, procrastination candidates, and non-procrastinators. Spectral clustering is used in this study (see Algorithm 2) to identify different clusters. In two clusters, Cluster A and B, with a small and rather large inactive times, and large and small spare time are considered as the non-procrastinators group (with a high average score) and procrastinators (with a low average score), respectively. In three clusters, Cluster A and C have the highest and lowest average score, smallest and largest inactive time, and largest and smallest spare time, respectively. Therefore, Cluster A is considered as non-procrastinators group and Cluster C as procrastinators. Cluster B, however, is regarded as procrastination candidate group with a medium average score (average = 61.45). Regarding four clusters, cluster A, B, and C appear to have a high average score, and cluster D has a very low average score. Cluster A and B with a small inactive time and bigger spare time compared to other clusters, and higher average scores can be considered as non-procrastinators. Cluster D, with the lowest average score, which has the largest inactive time and smallest spare time than the other three clusters, can be seen as procrastinators, while Cluster C with a relatively high average score, large inactive time, and small spare time can be called procrastination candidates. These findings suggest that the larger the average inactive time and the smaller the average of spare time is, the lower the average of assignment score of the students is. Additionally, this analysis indicates that no more distinct (significant) clusters can be achieved beyond three clusters. The Elbow method also implies that three clusters is the optimal number for our data. On the other hand, it is apparent that, for more personalization (personalized intervention), more clusters can be formed (a higher value for *k*) as the number of classes increases. Consequently, we conclude that two and four clusters of students result in either grouping procrastinators and procrastination candidates together (coarse clusters), or having some part of the procrastination candidates’ group shared with non-procrastinators (too detailed clusters), respectively, as our analysis showed the more clusters that are formed, the more groups of non-procrastinators emerge (reducing procrastination candidates). According to our analysis and the Elbow method, three clusters is the optimal number, which is well connected to the rationalization behind PPP (he correlation between spare time, inactive time, and average score of each cluster of students).

As a third step, we compared eight different classification methods, using four performance metrics of precision, accuracy, F1 score, and recall, to find the most suitable classifier for predicting students’ procrastination behaviors. According to our findings, the accuracy of the classification methods is almost higher with a smaller number of classes and all methods appear to be sensitive to the increment in the number of classes. In other words, the accuracy of the classification methods mostly increases with the decrement in class numbers. According to our findings, regarding the average of all performance metrics at different *k*-fold for all classification methods, among all classification methods, L-SVM and R-SVM are the best in two-class, at different *k*-fold, with continuous and categorical features, respectively. For three-class, L-SVM and NN are the best performed methods at different *k*-fold for continuous and categorical features, respectively. In four-class, NN mostly performs the best in both continuous and categorical feature at different *k*-fold.

As mentioned in previous sections, beyond three-class no significant group can be formed, therefore, we focus more on the comparison of the classification methods in three-class. It should be noted that in case educators intend to respond to students in a more personalized way, more classes could be considered in our proposed approach. A comparison of various metrics for three-class reveals that, for categorical features, NN outperforms other methods with precision, recall, accuracy, and F1-score of 95%, 96%, 96%, and 97%, respectively, whereas for continuous features L-SVM could achieve precision, recall, accuracy, and F1-score of 95%, outperforming other methods. Observe that by increasing the number of classes, some of these methods may perform slightly lower than others. In our approach, categorical features perform slightly better and more robust compared to continuous features. Furthermore, an increment in the number of classes results in a decrement of the prediction power of the classification methods. By employing different number of *k*-fold on the methods, we also found that, in our proposed approach, most of the methods tend to be rather stable using small or large *k*-folds. In other words, overall, classification methods show a slight increment or decrement of precision, accuracy, and F1-score at different *k*-folds, indicating the stability of our proposed approach.

Consequently, PPP proved to successfully predict students’ performance through their procrastination behaviors with an accuracy of 96%. Regarding the variable types used in feature vectors, we found categorical features to be more robust and perform slightly better than continuous features in PPP.

## 6. Conclusions

Students’ procrastination tendency is frequently named by several researchers as an important factor negatively influencing performance of students in online learning, making its prediction a very useful task for both educators and students. In this study, we proposed a novel algorithm, called PPP, which uses students’ assignment submission behavior to predict their performance through procrastination behavior. PPP, unlike existing works, not only considers late or non-submissions, but also investigates students’ behavioral patterns before a homework due date. The proposed approach, shown in Algorithm 3, is flexible and, in an automatic way, identifies students with different sorts of learning difficulties in various online learning environments as they mostly share the same characteristics as Moodle (e.g., they all mostly log opening, due, submission, and students’ first view date of the assignments and courses). To predict students’ procrastination tendencies, PPP firstly builds a feature vector representing the submission behavior of students for each assignment, using inactive and spare time. It then uses clustering and classification methods to put students into different procrastination behavior categories based on their submission behavior. A course including 242 students from the University of Tartu in Estonia was used to evaluate the effectiveness of PPP. The results reveal that in labelling students using clustering, two and four clusters of students results in either coarse clusters or too detailed clusters, as our analysis showed that the more clusters are formed, the more groups of non-procrastinators emerge (reducing procrastination candidates). As our analysis and the Elbow method show, three clusters is the optimal number, which is well connected to the rationalization behind PPP (correlation between spare time, inactive time, and average score of each cluster of students). Another important task was to classify students using the labels. In this phase, we compared eight different classification methods to find the most suitable classifier for predicting students’ procrastination behavior. Our findings in this regard show that the accuracy of the classification methods is almost higher with a smaller number of classes and all methods appeared to be sensitive to the increment in the number of classes. NN, with an accuracy of 96%, showed a better performance using categorical features compared to other classification methods, while L-SVM perform the best in continuous features with an accuracy of 95%. Finally, regarding the variable types used in the feature vectors, we found categorical features to be more robust and perform slightly better than continuous features. In conclusion, PPP could successfully predict students’ performance through their procrastination behavior with an accuracy of 96%.

In future work, we aim to extend our study by using feature vectors of different lengths from different courses. Additionally, we intend to create different hybrid feature vectors using students’ submission behavior to investigate the effect of various time-related indicators on the procrastination behavior of students.

## Figures and Tables

**Table 1:** Comparison of our proposed approach with related works.

	Objective	Behavioral Patterns before Submission		Attributes	Classification Techniques
		Inactive Time	Spare Time		
[17]	Prediction of assignment submission	no	no	-students’ activity data-course and assignment information	DT (CART), Random Forest, NN, GaussianNB, Logit, LDA, SVC
[50]	Prediction of students’ procrastination	no	yes	-grade	ZeroR, OneR, ID3, J48, Random Forest, Decision Stump, JRip, PART, NBTree, Prism
[51]	Prediction of students at risk through assignment submission	no	no	-students’ activity data-course and assignment information-peers activity data	Neural Network
Our work	Prediction of procrastination	yes	yes	-students’ activity and assignment data-grade	L-SVM, R-SVM, Gaussian Processes, Decision Tree, Random Forest, Neural Network, AdaBoost, Naive Bayes

**Table 2:** Notations.

Notation	Explanation
S, A	A set of students and assignments
s,a	A specific student and assignment
$v_{1},\;v_{2},\;w_{1},\;w_{2}$	A spare time and an inactive time (both continuous and categorical values)
$OpenD^{a}$	The open date of assignment
$Deadline^{a}$	The due date of assignment
$FirstviewD^{s}$	The student’s first view date of assignment
$SubmissionD^{s}$	The student’s assignment submission date
$x_{i}$,$\;y_{i}$	A pair of continuous and categorical features for an assignment *i*, $x_{i}\;=\;\left(v_{1},\;v_{2}\right),\;\;y_{i\;}=\;\left(w_{1},\;w_{2}\right)$
$X_{j}$, $Y_{j}$	Continuous and categorical feature vectors for a student *j*, $X_{j}\;=\;(x_{1j},\;x_{2j},\ldots x_{ij})$*,* $Y_{j}\;=\;\left(y_{1j},\;y_{2j},\ldots y_{ij}\right)$
**W**	Weighted adjacency matrix
L	Unnormalized Laplacian
**u**	Eigenvector
**U**	The matrix containing the eigenvectors
*P*	The set of Performance metrics
*C*	The best classification method

**Table 3:** Datasets used in this study.

	Course	Period	Type	# of Assignments	# of Students
Dataset 1 (16 continuous features)	Teaching and reflection	2019	blended	8	242
Dataset 2 (16 categorical features)	Teaching and reflection	2019	blended	8	242

**Table 4:** Statistical analysis.

	$\mathrm{Spare}\;\mathrm{Time}\;(v_{1})$	$\mathrm{Inactive}\;\mathrm{Time}\;(v_{2})$	Score
spare time ($v_{1}$)	1	–0.495	0.901
inactive time ($v_{2}$)	–0.495	1	–0.508
score	0.901	–0.508	1
count	242	242	242
mean	7.185	4	80.902
standard deviation	1.867	2.578	24.579
minimum	0	0	–3.333
maximum	8	8	100

**Table 5:** Performance metrics for all classification methods.

**Cluster 2**	**L-SVM**	**R-SVM**	**GP**	**DT**	**RF**	**NN**	**ADB**	**NB**
**Continuous Features**								
Precision	0.992	0.974	0.981	0.983	0.983	0.985	0.987	0.985
Recall	0.993	0.980	0.984	0.985	0.985	0.986	0.986	0.984
Accuracy	0.993	0.980	0.984	0.985	0.985	0.986	0.986	0.984
F1-score	0.993	0.982	0.986	0.986	0.986	0.987	0.988	0.986
**Categorical Features**								
Precision	0.984	0.992	0.989	0.991	0.990	0.990	0.990	0.989
Recall	0.992	0.996	0.994	0.996	0.995	0.994	0.994	0.994
Accuracy	0.992	0.996	0.994	0.996	0.995	0.994	0.994	0.994
F1-score	0.996	0.998	0.997	0.998	0.997	0.997	0.997	0.997
**Cluster 3**	**L-SVM**	**R-SVM**	**GP**	**DT**	**RF**	**NN**	**ADB**	**NB**
**Continuous Features**								
Precision	0.957	0.934	0.943	0.935	0.935	0.940	0.931	0.892
Recall	0.952	0.929	0.937	0.927	0.926	0.933	0.920	0.885
Accuracy	0.952	0.929	0.937	0.927	0.926	0.933	0.920	0.885
F1-score	0.952	0.930	0.938	0.929	0.928	0.935	0.922	0.886
**Categorical Features**								
Precision	0.867	0.920	0.938	0.946	0.950	0.952	0.954	0.956
Recall	0.930	0.954	0.961	0.963	0.963	0.965	0.963	0.962
Accuracy	0.930	0.954	0.961	0.963	0.963	0.965	0.963	0.962
F1-score	0.963	0.970	0.974	0.975	0.974	0.975	0.973	0.973
**Cluster 4**	**L-SVM**	**R-SVM**	**GP**	**DT**	**RF**	**NN**	**ADB**	**NB**
**Continuous Features**								
Precision	0.764	0.809	0.862	0.861	0.862	0.877	0.850	0.813
Recall	0.842	0.841	0.880	0.868	0.868	0.881	0.837	0.805
Accuracy	0.842	0.841	0.880	0.868	0.868	0.881	0.837	0.805
F1-score	0.899	0.874	0.903	0.888	0.885	0.896	0.852	0.820
**Categorical Features**								
Precision	0.596	0.778	0.843	0.856	0.858	0.866	0.848	0.855
Recall	0.719	0.840	0.887	0.886	0.881	0.889	0.870	0.873
Accuracy	0.719	0.840	0.887	0.886	0.881	0.889	0.870	0.873
F1-score	0.788	0.874	0.911	0.905	0.898	0.905	0.889	0.891

## Appendix A

As mentioned in previous sections, beyond three-class no significant group can be formed, therefore, we only present, in Table A1, performance metrics of classification methods in three-class at different *k*-folds.

**Table A1:** Performance metrics of classification methods in three-class at different *k*-folds (i.e., 5, 10, 15, and 20).

**Three-Class**	**L-SVM**	**R-SVM**	**GP**	**DT**	**RF**	**NN**	**ADB**	**NB**
**Continues Features**								
Precision_5	0.958	0.933	0.940	0.933	0.933	0.939	0.927	0.898
Precision_10	0.963	0.937	0.944	0.933	0.934	0.938	0.930	0.899
Precision_15	0.952	0.934	0.945	0.941	0.940	0.944	0.937	0.893
Precision_20	0.954	0.934	0.943	0.933	0.934	0.940	0.930	0.879
**Categorical Features**								
Precision_5	0.867	0.923	0.941	0.948	0.952	0.955	0.957	0.958
Precision_10	0.866	0.913	0.934	0.941	0.944	0.945	0.949	0.949
Precision_15	0.868	0.923	0.937	0.944	0.951	0.953	0.955	0.957
Precision_20	0.868	0.921	0.941	0.951	0.953	0.955	0.957	0.959
**Three-Class**	**L-SVM**	**R-SVM**	**GP**	**DT**	**RF**	**NN**	**ADB**	**NB**
**Continues Features**								
Accuracy_5	0.950	0.924	0.932	0.926	0.926	0.934	0.918	0.884
Accuracy_10	0.959	0.930	0.938	0.927	0.926	0.931	0.920	0.885
Accuracy_15	0.946	0.932	0.941	0.932	0.931	0.937	0.927	0.890
Accuracy_20	0.951	0.930	0.935	0.923	0.922	0.928	0.916	0.881
**Categorical Features**								
Accuracy_5	0.929	0.954	0.961	0.961	0.961	0.963	0.962	0.962
Accuracy_10	0.930	0.951	0.959	0.962	0.961	0.962	0.960	0.960
Accuracy_15	0.930	0.955	0.960	0.962	0.963	0.965	0.963	0.962
Accuracy_20	0.929	0.954	0.963	0.967	0.966	0.968	0.965	0.963
**Three-Class**	**L-SVM**	**R-SVM**	**GP**	**DT**	**RF**	**NN**	**ADB**	**NB**
**Continues Features**								
F1_5	0.949	0.922	0.931	0.925	0.926	0.933	0.917	0.874
F1_10	0.958	0.929	0.937	0.928	0.927	0.932	0.921	0.881
F1_15	0.948	0.935	0.944	0.936	0.935	0.940	0.929	0.895
F1_20	0.953	0.934	0.938	0.927	0.926	0.933	0.921	0.894
**Categorical Features**								
F1_5	0.963	0.969	0.973	0.971	0.972	0.973	0.970	0.970
F1_10	0.963	0.969	0.974	0.974	0.972	0.974	0.972	0.972
F1_15	0.963	0.971	0.972	0.973	0.974	0.975	0.973	0.973
F1_20	0.962	0.972	0.979	0.979	0.978	0.979	0.978	0.977
